# Variation in somatic symptoms by patient health questionnaire-9 depression scores in a representative Japanese sample

**DOI:** 10.1186/s12889-018-6327-3

**Published:** 2018-12-27

**Authors:** Eri Hoshino, Sachiko Ohde, Mahbubur Rahman, Osamu Takahashi, Tsuguya Fukui, Gautam A. Deshpande

**Affiliations:** 1 Graduate School of Public Health, Center for Clinical Academia, 5th Floor, St. Luke’s International University, Tsukiji 3-6-2, Chuou-ku, Tokyo, 104-0045 Japan; 2 St. Luke’s International Hospital, Tokyo, Japan

**Keywords:** Depression, PHQ-9, Somatic symptoms, Health dairy, Japan, Self-report

## Abstract

**Background:**

This study aims to evaluate variation in somatic symptoms by age using patient health questionnaire-9 (PHQ) depression scores, which may be helpful in identifying depression.

**Methods:**

The study evaluated a nationally representative cross-sectional sample of community-dwelling adults in Japan in 2013. We utilized the PHQ to identify risk for depression, with PHQ ≥ 10 defining at least moderate depression. Bivariate and factor analyses were used to capture underlying patterns in self-reported symptoms over a 30 day period; aged-stratified multivariate logistic regression was performed to further explore associations between age, symptoms, and depression.

**Results:**

Of 3753 respondents, 296 (8, 95% CI 7.0–8.8) reported a PHQ ≥ 10; 42% of these were male and mean age was 51.7 years old (*SD* = 18.6). Multivariate analysis showed that presence of fatigue and malaise (OR = 1.7, 95% CI 1.3–2.4) was significantly associated with PHQ ≥ 10. After stratification by age, PHQ ≥ 10 was associated with gastrointestinal complaints among 18–39 year olds (OR = 1.7, 95% CI 1.0–2.9); fatigue and malaise (OR = 1.8, 95% CI 1.1–3.1) among 40–64 year olds; and fatigue and malaise (OR = 1.8, 95% CI 1.1–3.0) as well as extremity pain (OR = 1.7, 95% CI 1.0–2.8) in over 65 year olds.

**Conclusion:**

Age-related somatic symptom correlates of PHQ ≥ 10 differ across the lifespan. Predominantly gastrointestinal symptoms in younger patients, and generalized fatigue, malaise, and musculoskeletal pain in older groups were observed. In order for screening physicians to proactively identify depression, awareness of age-related somatic symptoms is warranted.

## Background

By 2030, depressive disorders are estimated to overtake ischemic heart disease as the second leading cause of disability worldwide; in high income countries, depression is predicted to be the leading cause of disability [1]. Though depression has been associated with an overall 4% lifetime suicide risk [2], 18.5 individuals out of every 100,000 committed suicide in Japan in 2012, a rate roughly 60% higher than the global average, and the 5th highest globally [3]. In contrast to the high incidence of suicide, previous literature has consistently reported a lower prevalence of depression in Japan, ranging from 2 to 6% [4–6]. This is substantially lower compared to other Western countries such as the United States, with a reported 16% prevalence [7], and strongly suggests the possibility of underreporting and underdiagnoses of clinically significant depression in Japan.

Per previous reports, patients with somatic symptoms have a greater risk of developing depression [8–10]; two-thirds of primary care patients with depression present with somatic symptoms, though the typically wide spectrum, which includes fatigue, changes in sleep and appetite, headache, back problems, and chronic pain, makes early detection of depression particularly challenging [11–13]. Predictive characteristics of somatic symptoms that may reflect depression, and the pattern by which these change with age and other social factors, has not been fully investigated using representative population-level data.

Previous studies from a variety of countries have also identified an association between depression and sociodemographic and socioeconomic factors. Multiple studies have shown a higher prevalence of depression among women compared to men, suggesting a differing pathophysiology by sex [14]. Major depression also differs by age, with previous studies reporting that depression was less common in older community-dwelling adults [15, 16]. Previous studies done in Western countries suggest that low SES is also associated with psychological problems including depression, anxiety, and mood disorders [17, 18] . However, the literature suggests that these associations vary substantially by country; for instance, while education has been found to be inversely associated with depression in the United States, no such association has been found in Japan [19]. Previous literature has also demonstrated the relationship between depression and past history of depression, both personal and in family members [20], as well as with comorbidities including cardiac disease, diabetes, cancer, pain, and obesity [21].

Given the relatively high incidence of suicide, we hypothesize that depression is far more prevalent in Japan than has been previously reported. In addition, sociodemographic and socioeconomic patterns related to depression seen in other countries might differ in Japan. This study aims to evaluate patterns of somatic symptoms, which may be helpful in identifying depression, vary with age and other sociodemographic, socioeconomics and health-related factors.

## Methods

This cross-sectional investigation was part of the Health Diary Study, a national-level exploration of Japan’s health ecology, conducted in October 2013 among community-dwelling residents across Japan [22]. Consisting of a baseline questionnaire and a 31-day symptom diary, the Health Diary Study aimed to first capture the baseline demographic, socioeconomic, health status, and lifestyle habits of respondents, followed by an immediate and continuous record of daily health events and care-seeking behavior over the ensuing month [23]. A population-weighted random sample of households was drawn from a panel of survey respondents provided by a commercial survey company (JMA Ltd., Tokyo, Japan).

### Participants and procedure

Of a panel of approximately 80,000 individuals, 19,633 agreed to participate in the Health Diary Study. From this group, 5000 participants were randomly selected for inclusion of the study. Informed consent was obtained through documents sent by mail and $30 gift voucher was given to each participant. Participants were asked to answer a self-administered questionnaire consisting of baseline information on demographic, socioeconomic, current and previous health status, and lifestyle habits including smoking and alcohol consumption. Participants were given a weekly reminder to complete their health diaries via phone.

### Depression measure

The Patient Health Questionnaire (PHQ-9) portion of the survey was used to measure depression. The PHQ-9 is a well-validated, Diagnostic and Statistical Manual of Mental Disorders- Fourth Edition (DSM-IV) criterion-based measure for diagnosing depression, assessing severity and monitoring treatment response [24]. The PHQ-9 score can range from 0 to 27, with each of the 9 items scored on a 0 (not at all) to 3 (nearly every day) scale. A PHQ score of ≥10 has been reported to have both a sensitivity and specificity for major depression of 88% [25]; the Japanese language version has been previously validated in Japan [26]. Our primary outcome of interest was “at least moderate depression”, reflected by a PHQ score of ≥10—the most commonly accepted reasonable threshold that we consider warranting follow-up screening and identification [25].

### Self-reported symptoms

A subsequent health diary questionnaire was provided, asking participants to record self-reported symptoms, daily health-related events, and health behaviors related to each event for 31 consecutive days. Total number of symptoms reported, as well as types of symptoms, were recorded.

### Sociodemographic, socioeconomic, and health-related variables

Variables included age, gender, population of area of residence (≥ 100,000 residents), marital status (married, never-married, widowed, and divorced), occupational status (full-time, part-time, jobless, and others including homemakers, retirees, and students), educational attainment (less than high school, vocational, two-year college, and above college level), and household income (less than $30,0000; $30,000–$60,000; $60,000–$100,000; and above $100,000; currency was adjusted as 1$ = 100 yen). Health-related characteristics included obesity which is generally considered as body mass index (BMI) ≥ 30, though in our study, we used a stricter obesity criteria of BMI ≥ 25 per World Health Organization Asia guidelines [27]. Additional characteristics included smoking status, excessive alcohol consumption (> 21 and > 14 units per week for men and women, respectively; one unit of alcohol is equivalent to 20 g of pure alcohol) [28], having a primary care physician (PCP), family history of depression, and prior diagnosis of depression. Data on comorbidities were included based on those diseases linked to depression in previous studies, including prior diagnosis of cardiac disease, cerebrovascular disease, Alzheimer’s disease, epilepsy, diabetes, and cancer [21].

For age-stratified analysis, the sample was categorized into three age groups: 18–39 years old (young adult), 40–64 years old (middle-aged), and > 65 years old (elderly) based on definitions for working stage of life applied from the White Paper on National Lifestyle [29] and the Ministry of Health, Labor, and Welfare of Japan [30]. We used age classifications that were roughly equivalent to previous international classifications [31]: 15–24 years old (youth), 25–44 years old (young adulthood), 45–64 years old (middle adulthood), and > 65 years old (older adulthood). As our sample included only those 18 years and above, the two youngest age groups were combined to maintain statistical power.

### Statistical methods

Descriptive statistics describing participants’ sociodemographic, socioeconomic status, and health-related characteristics were generated. Comparing those participants identified as at risk for at least moderate depression (PHQ ≥ 10) to those without, we compared the data using t-test or Mann-Whitney’s U test for continuous variables and Chi-squared test for categorical variables. For analysis of self-reported symptoms, factor analysis was conducted using Varimax rotation to categorize symptoms by domains. Factor analysis is a method for modeling observed variables in terms of a smaller number of underlying unobservable domains. Since our self-reported symptoms varied greatly in both type and frequency, factor analysis facilitated the identification of interpretable narrow-band factors. We conducted a subsequent multivariate logistic regression controlling for all relevant covariates. We included all predictors significant at the 0.1 significance value or less and clinically meaningful variables for age-stratified analysis in order to investigate the association between self-reported symptoms and PHQ ≥ 10 at different periods of life. All statistical analyses were performed using Stata version 14.1 (Stata-Corp, College Station, TX).

### Ethics and consent to participate

Ethics approval was obtained from the Research Ethics Committee of St Luke’s International University (#15-R142). Written informed consent was obtained from participants prior to conducting the study.

## Results

### Characteristics of participants

The response rate was 91% (*n* = 4548) and 81% (*n* = 4039) for the baseline questionnaire and health diary, respectively. Our analysis included participants who were at least 18 years old and completed the PHQ-9 (*n* = 3753), excluding those participants with incomplete PHQ-9 data (*n* = 34). Among those with incomplete PHQ-9 data, 18 provided no PHQ-9 data. Of the 16 who provided partial responses, 4 had scored ≥10 despite providing incomplete surveys. PHQ-9 data was categorized according to the previously definition of depression severity. For scores of 0–4 (none), 5–9 (mild), 10–14 (moderate), 15–19 (moderately severe), and ≥ 20 (severe), there were 2729 (73%), 728 (19%), 187 (5%), 83 (2%), and 26 (1%) individuals, respectively. Descriptive statistics on participants are presented in Table 1. A total of 296 individuals (8, 95% CI 7.0–8.8) reported a PHQ score of ≥10. Of all included respondents, the mean age was 51.7 (*SD* = 18.6) years old and 1777 (47%) were men. 2791 (74%) resided in an area with a population above 100,000, and 2463 (68%) were married. 1593 (43%) worked full-time, 1580 (42%) had a high school degree or less, and 1628 (43%) earned an income between $30,000 and $60,000.

**Table 1:** Participants’ characteristics and comparison by PHQ score

Variables		All (*n* = 3753) n(%)	PHQ ≥ 10 (*n* = 296) n(%)	PHQ < 10 (*n* = 3457) n(%)	*p*
1A: Sociodemographic & Socioeconomic Status					
Age (years), M (*SD*)					
18–39		1149 (30.6)	99 (33.4)	1050 (30.3)	<.01
40–64		1552 (41.4)	93 (31.4)		
≥ 65		1052 (28.0)	104 (35.2)	1459 (42.2)	
				948 (27.5)	
Gender, male		1777 (47.4)	125 (42.2)	1652 (47.8)	.06
Residence population > 100,000 persons		2791 (74.4)	221 (74.7)	2570 (74.3)	.90
Marital status (*n* = 3691)					
Married		2463 (66.7)	172 (58.7)	2291 (67.2)	<.01
Never-married		868 (23.5)	75 (25.4)	793 (23.4)	
Widowed		250 (6.8)	36 (12.2)	214 (6.4)	
Divorced		110 (3.0)	9 (3.7)	101 (3.0)	
Occupational status (*n* = 3693)					
Full-time		1593 (43.1)	93 (32.4)	1500 (44.0)	< 0.01
Part-time		552 (15.0)	46 (16.0)	506 (14.9)	
Homemaker		577 (15.6)	39 (13.6)	538 (15.8)	
Retiree		575 (15.6)	61 (21.2)	514 (15.1)	
Jobless		214 (5.8)	38 (13.2)	176 (5.2)	
Student		182 (4.9)	10 (3.5)	172 (5.0)	
Educational attainment (*n* = 3695)					
≤ High school		1580 (42.8)	150 (50.7)	1430 (41.4)	<.01
Two-year college		871 (23.6)	62 (21.3)		
College or higher		1244 (33.6)	79 (27.2)	809 (23.8)	
				1165 (34.2)	
Household income ($10,000)					
< 3		504 (13.4)	68 (23.0)	436 (12.6)	<.01
≥ 3 < 6		1628 (43.4)	116 (39.2)	1512 (43.7)	
≥ 6 < 10		1201 (32.0)	85 (28.7)	1116 (32.3)	
≥ 10		420 (11.2)	27 (9.1)	393 (11.4)	
1B: Health-related Characteristics					
At least one self-reported symptom		2973 (78.5)	267 (89.0)	2706 (77.6)	<.01
Mean number of self-reported symptom (*SD*)		12.1 (19.7)	25.5 (32.5)	11.0 (17.7)	<.01
Obese (BMI ≥ 25), (*n* = 3749)		724 (19.3)	77 (26.1)	647 (18.7)	.02
Currently smoking		495 (13.2)	40 (13.5)	455 (13.1)	.86
Having a primary care physician (*n* = 3741)		1890 (50.5)	190 (64.6)	1700 (49.3)	<.01
Family history of depression		90 (2.4)	12 (4.0)	78 (2.3)	.05
Prior diagnosis of depression		98 (2.6)	39 (13.2)	59 (1.7)	<.01
Prior diagnosis of depression related diseases		316 (8.4)	52 (17.6)	264 (7.6)	<.01
Excessive alcohol		98 (2.6)	10 (3.4)	88 (2.6)	.39

Note. *PHQ* Patient Health Questionnaire, scores ≥10 defining at least moderate depression; *BMI* Body Mass Index; Chi-square and student t-tests were used for categorical and continuous data, respectively

For health-related characteristics, 2973 (79%) reported at least one symptom during the 31 days of the health diary portion of the study. The mean number of symptoms reported during study participation was 12.1 (±19.7). 724 (19%) respondents had a BMI ≥ 25, 495 (13%) were current smokers, 1890 (51%) reported having a PCP, 98 (3%) had a prior diagnosis of depression, and 90 (2%) had a family history of depression. Among all, 316 individuals (8%) had a prior diagnosis of a comorbidity associated with depression, and 98 (3%) reported excessive alcohol consumption.

### Comparison of sociodemographic, socioeconomic, and clinical characteristics by PHQ score

The results of comparisons of sociodemographic, socioeconomic, and clinical characteristics in relation to PHQ ≥ 10 are summarized in Table 1. 89% of people with PHQ ≥ 10 reported at least one symptom while 78% of those without depression reported at least one symptom (*p* < 0.01). The mean number of symptoms per month was also markedly higher in those with PHQ ≥ 10 at 25.5 (*SD* = 32.5) versus 11.0 (*SD* = 17.7) (*p* < .01); there was a positive linear relationship between number of symptoms and PHQ score. PHQ ≥ 10 was also associated with older age, less full-time work, lower educational status, and lower income (*p* < .01 for all). 26% of people with PHQ ≥ 10 were obese, compared to 19% of those with PHQ < 10 (*p* = .02). 65% of people with PHQ ≥ 10 reported having a PCP versus 49% of those with PHQ < 10 (*p* < .01). 4% of people with PHQ ≥ 10 reported a family history of depression compared to 2% of those with PHQ < 10 (*p* = .05). 18% of people with PHQ ≥ 10 had a comorbid disease versus 8% of those with PHQ < 10 (*p* < .01).

### Factor analysis of self-reported symptoms by PHQ score

The frequency of self-reported symptoms between those reporting PHQ ≥ 10 versus < 10 are shown in Table 2. Headache was the most frequently reported symptom in both groups, although the frequency was higher in those with PHQ ≥ 10 (42% vs. 36%, *p* = .10). Other symptoms commonly reported in both groups included low back pain, neck and shoulder pain, generalized weakness and malaise, fever, and foot pain.

**Table 2:** Frequency of self-reported symptoms by PHQ score

At least moderate depression (PHQ ≥ 10), *n* = 267		No depression (PHQ < 10), *n* = 2706	
Symptoms	n, (%)	Symptoms	n, (%)
Headache	112 (41.9)	Headache	967 (35.7)
Low back pain	100 (37.5)	Sneezing	932 (34.4)
Generalized weakness/malaise	70 (26.2)	Low back pain	907 (33.5)
Sneeze	63 (23.6)	Sore throat	602 (22.2)
Neck and shoulder discomfort	61 (22.8)	Cough	554 (20.5)
Generalized abdominal pain/severe pain	46 (17.2)	Neck and shoulder discomfort	523 (19.3)
Cough	44 (16.5)	Generalized weakness/malaise	467 (17.3)
Sore throat	40 (15.0)	Generalized abdominal pain/severe pain	384 (14.2)
Fever	33 (12.4)	Knee pain	286 (10.6)
Stomach ache	32 (12.0)	Fever	281 (10.4)
Foot pain/toe pain	30 (11.2)	Shoulder pain	258 (9.5)
Knee pain	29 (10.9)	Foot pain/toe pain	245 (9.1)
Nausea	24 (9.0)	Myalgia	224 (8.3)
Clumsiness/sleepiness/site-unspecified pain	22 (8.2)	Diarrhea	213 (7.9)

Note. *PHQ* Patient Health Questionnaire

In order to better understand and identify underlying patterns in self-reported symptoms, we conducted an exploratory factor analysis. Analysis of symptoms among those with PHQ ≥ 10 yielded five interpretable factors demonstrating eigenvalues greater than 0.9 and accounting for 62% of the total variance (Appendix 1 and 2). The five-factor structure was constructed based on the largest item-factor loading, which exceeded 0.40. The first factor included neck and upper shoulder discomfort (*r* = .61), shoulder pain (*r* = .57), headache (*r* = .50), and low back pain (*r* = .47). The second factor included cough (*r* = .73), fever (*r* = .63), and sore throat (*r* = .61). The third factor included generalised abdominal pain (*r* = .74) and diarrhea (*r* = .71). The fourth factor contained fatigue and somnolence (*r* = .79) and general weakness and malaise (*r* = .70). The fifth factor included knee pain (*r* = .67) and foot pain (*r* = .48). The most satisfactory grouping of self-reported symptom was constructed as follows: cervicolumbar musculoskelatal pain (Domain 1), upper respiratory symptoms (Domain 2), gastrointestinal complaints (Domain 3), fatigue and malaise (Domain 4), and extremity pain (Domain 5).

We compared the frequency of each of these five domains among those reporting PHQ ≥ 10 versus < 10 (Table 3). All symptom domains were more frequently observed in those with PHQ ≥ 10. Cervicolumbar musculoskelatal pain was observed in 60% of individuals with PHQ ≥ 10 versus 50% in individuals with PHQ < 10; upper respiratory symptoms were observed in 29% versus 22%; gastrointestinal complaints were observed in 19% versus 11%; fatigue and malaise was observed in 27% versus 14%; and extremity pain was observed in 19% versus 12% (*p* < 0.01 for all comparisons).

**Table 3:** Comparison of major symptoms by PHQ score

Symptom Domain	All (*n* = 3753)	At least moderate depression (PHQ ≥ 10), *n* = 296	No depression (PHQ < 10), *n* = 3457	*p*
Cervicolumbar musculoskeletal pain, n (%)	1917 (51.1)	177 (59.8)	1740 (50.3)	.01
Upper respiratory symptoms, n (%)	848 (22.6)	87 (29.4)	761 (22.0)	.01
Gastrointestinal complaints, n (%)	448 (11.9)	55 (18.6)	393 (11.4)	<.01
Fatigue and malaise, n (%)	562 (15.0)	79 (26.7)	483 (14.0)	<.01
Lower extremity pain, n (%)	463 (12.3)	56 (18.9)	407 (11.8)	<.01

Note. Chi-squared tests were used for group comparisons

*PHQ* Patient Health Questionnaire

### Factors associated with PHQ ≥ 10

The results of our multivariate analysis for those with PHQ ≥ 10, adjusting for sociodemographic, socioeconomic, health-related, and symptom characteristics, are shown in Table 4. For sociodemographic and socioeconomic characteristics, using those < 39 years old as reference, individuals 40 to 64 years old were more likely to have PHQ ≥ 10 (OR = 1.83, 95% CI, 1.13–2.96). Though there was a trend towards PHQ ≥ 10 in elderly women, this did not reach the level of statistical significance (OR = 0.65, *p* = .07) and was certainly less robust than the risk for middle-age Japanese men. Compared to those with full-time employment, jobless individuals were more than twice as likely to report PHQ ≥ 10 (OR = 2.38, 95% CI 1.44–3.92). In our sub-analysis, this relationship is true for men (OR = 2.83, 95% CI 1.42–5.67) but not for women (OR = 1.96, 95% CI 0.95–3.98). Educational attainment above college level showed a non-significant trend toward less depression (OR = 0.74, 95% CI 0.53–1.04) compared to those attaining less than a high school degree. In comparison with annual household incomes <$30,000, those with incomes $30,000 -$60,000 were less likely to report depression (OR = 0.58, 95% CI 0.40–0.85); though a general trend of decreasing depression with increasing income was seen, a statistically significant linear relationship was not identified for higher income brackets.

**Table 4:** Risk of depression in patients with versus without PHQ ≥ 10

Variables	All (*n* = 3753)
	OR (95% CI)
Sociodemographic & Socioeconomic Status	
Age	
18–39	–
40–64	1.83 (1.13–2.96)
≥ 65	0.82 (0.54–1.23)
Gender, male	1.05 (0.76–1.44)
Residence population > 100,00 people	1.06 (0.79–1.44)
Marital status	
Married	–
Never-married	0.86 (0.58–1.27)
Widowed	1.21 (0.74–1.98)
Divorced	0.91 (0.43–1.93)
Occupational status	
Full-time	–
Part-time	1.13 (0.74–1.73)
Jobless	2.38 (1.44–3.92)
Others (Homemaker, Retiree, Student)	1.11 (0.76–1.63)
Educational attainment	
≤ High school	–
Two-year college	0.81 (0.57–1.14)
College or higher	0.74 (0.53–1.04)
Household income ($)	
< 30,000	–
≥ 30,000 < 60,000	0.58 (0.40–0.85)
≥ 60,000 < 100,000	0.69 (0.46–1.02)
≥ 100,000	0.61 (0.36–1.04)
Health-related Characteristics	
Obese (BMI ≥ 25)	1.42 (1.04–1.93)
Currently smoking	1.09 (0.72–1.64)
Having a primary care physician	1.36 (1.01–1.82)
Family history of depression	1.56 (0.78–3.13)
Prior diagnosis of depression	6.11 (3.77–9.89)
Prior diagnosis of depression related comorbidity	2.14 (1.47–3.12)
Excessive alcohol	1.62 (0.78–3.36)
Symptom Domain	
Cervicolumbar musculoskeletal pain	1.07 (0.80–1.42)
Upper respiratory symptoms	1.06 (0.79–1.44)
Gastrointestinal complaints	1.38 (0.97–1.98)
Fatigue and malaise	1.73 (1.26–2.37)
Pain in lower extremity	1.21 (0.84–1.74)
Mean number of self-reported symptom	1.02 (1.01–1.02)

Note. *PHQ* Patient Health Questionnaire, scores ≥10 defining at least moderate depression; *BMI* Body Mass Index

For health-related characteristics, obesity (OR = 1.42, 95% CI 1.04–1.93), having a PCP (OR = 1.36, 95% CI 1.01–1.82), prior diagnosis of depression (OR = 6.11, 95% CI 3.77–9.89), and prior diagnosis of medical comorbidities (OR = 2.14, 95% CI 1.47–3.12) demonstrated a statistically significant association with PHQ ≥ 10. For symptom domains identified in the factor analysis, fatigue and malaise, Domain 4, (OR = 1.73, 95% CI 1.26–2.37) and number of self-reported symptoms in a month (OR = 1.02, 95% CI 1.01–1.02) were also positively associated with PHQ ≥ 10. Gastrointestinal complaints, Domain 3, demonstrated a trend towards PHQ ≥ 10 (OR = 1.38, 95% CI 0.97–1.98). Other symptom domains, including cervicolumbar pain, extremity pain, and upper respiratory symptoms, were not statistically associated with PHQ ≥ 10 after adjustment for covariates.

The results of our age-stratified multivariate regression controlling for gender, household income, history of depression and depression-associated comorbidities, and number of symptoms per month are shown in Table 5. Gastrointestinal complaints, Domain 3, were positively associated with PHQ ≥ 10 (OR = 1.71, 95% CI 1.01–2.91) only among those 18 to 39 years old, while in the 40–64 year old cohort, fatigue and malaise, Domain 4, were associated with higher probability of PHQ ≥ 10 (OR = 1.82, 95% CI 1.08–3.05). In the elderly cohort, fatigue and malaise, Domain 4, (OR = 1.80, 95% CI 1.07–3.02) as well as extremity pain, Domain 5, (OR = 1.69, 95% CI 1.03–2.79) demonstrated a significant association with PHQ ≥ 10.

**Table 5:** Risk of at least moderate depression by somatic symptom type

Symptom Domain	Age 18–39 *n* = 1149		Age 40–64 *n* = 1552		Age Above 65 *n* = 1052	
	OR (95% CI)	*p*	OR (95% CI)	*p*	OR (95% CI)	*p*
Cervicolumbar musculoskeletal pain	1.12 (0.69–1.82)	.64	1.16 (0.70–1.91)	.57	0.86 (0.54–1.38)	.54
Upper respiratory symptoms	0.76 (0.45–1.28)	.31	0.93 (0.55–1.56)	.78	1.46 (0.88–2.41)	.15
Gastrointestinal complaints	1.71 (1.01–2.91)	.05	1.24 (0.69–2.23)	.48	0.94 (0.41–2.14)	.88
Fatigue and malaise	1.29 (0.71–2.36)	.40	1.82 (1.08–3.05)	.02	1.80 (1.07–3.02)	.03
Pain in lower extremity	1.09 (0.43–2.77)	.85	0.72 (0.37–1.38)	.32	1.69 (1.03–2.79)	.04
At least moderate depression (PHQ ≥ 10), n (%)	99 (8.62)		93 (5.99)		104 (9.89)	

Note. Controlled for gender, household income, having a primary care physician, history of depression, history of depression-related comorbidity, and number of symptoms per month; *PHQ* Patient Health Questionnaire, scores ≥10 defining at least moderate depression

## Discussion

This study evaluated the variation in somatic symptoms among community-dwelling adults in Japan by PHQ depression scores, further exploring associations between age, symptoms, and depression. At 8%, the prevalence of PHQ ≥ 10 in Japan, defined as PHQ ≥ 10, is lower than that previously reported in Western countries [32], though slightly higher than previous reports from Japan [5, 6]. Interestingly, among those reporting PHQ ≥ 10 in our study, only 13% (39/296) had been ever diagnosed as having depression, suggesting substantial underdiagnosis of depression in the Japanese population. Similarly, among those reporting severe depression (PHQ ≥ 15), only 21% (23/109) carried a previous diagnosis of depression. We hypothesize that the possible underdiagnosis in Japan might be related to the persistent cultural stigma surrounding mental disorders. Stigmatizing attitudes towards mental disorders continue to exist among the general population, and may be more prevalent when compared to Western countries; this may reduce the likelihood of seeking professional help and support, resulting in underdiagnosis [33, 34].

Only 65% of patients with PHQ ≥ 10 reported having a PCP. This suggests that the underutilization of primary care physicians in the mental health screening process in Japan may play a critical role in underdiagnosis. Reports from North America suggest that up to 42% of clinical depression is identified and treated in the primary care setting [35]. As the role of primary care physicians in comprehensive and preventive care is substantially less well-established in Japan [36], this study identifies a potentially important area of further development for primary care specialists.

Our findings are consistent with those of previous literature in that patients with somatic symptoms have a greater risk for depression [8–10]. After controlling for sociodemographic, socioeconomic characteristics, and clinical characteristics, the number of self-reported symptoms per month remained statistically significant in the group with PHQ ≥ 10. Analyses of specific symptoms suggest that certain symptoms may be more highly related to depression, with previous literature suggesting and association with fatigue and insomnia [37], fatigue, musculoskeletal complaints, back pain, unexplained physical symptoms [38], and gastrointestinal disorders. Another study related insomnia, fatigue, chronic pain, fair or poor self-rated health, and unexplained physical symptoms with depression [39]. The result of our adjusted multivariate analysis corroborate the statistical association of fatigue and/or malaise with PHQ ≥ 10. Interestingly, however, our age-stratified multivariate logistic regression showed that when predicting depression, symptoms may differ substantially by age. For those 18 to 39 years old, only presence of gastrointestinal symptoms was associated with PHQ ≥ 10, while fatigue and malaise began to show an association only after 40 years old. In addition to fatigue, among those older than 65 years, extremity pain also showed an association with higher probability of depression. These findings may belie a pathophysiologic basis of depression rooted in disturbance of serotonin and other neurotransmitters important for gastrointestinal functioning, as has been suggested in both animal and human models [40, 41], among younger individuals, while suggesting that degenerative disorders and their relationship to overall failing vitality may play a larger role in mental health as age increases.

Our study failed to identify a statistically significant association between gender and higher probability of depression after adjustment for relevant covariates. This finding contradicts those of previous studies from several countries, which have reported a higher prevalence of depression among women [42, 43]. These results suggest that gender may be associated with culture-specific drivers of depression.

Our multivariate regression showed that joblessness was markedly more associated with depression risk compared to full-time employment; this may further highlight the importance, and perhaps over-emphasis, of the important role of work in Japanese society. The well-known Japanese cultural predilection for commitment to employed work, with perhaps overwork or low job satisfaction, may be particular drivers for depression; 30% of male workers reported working 49 h or more per week in 2014, despite national law defining a normal work week as 40 h [44]. Moreover, the mean age of onset of overwork-related disorders--including *karoshi*, a medicolegally established concept in Japan of death related directly to overwork--was 49.3 (*SD* = 9.7) [45]. Interestingly, our data suggests that the importance of work in a Japanese individual’s life, at least as it relates to risk for depression, may not be intimately tied to income. As compared to incomes of less than $30,000, higher incomes were negatively associated with depression, but the relationship did not meet statistical significance beyond a threshold of $60,000. In various previous studies, depression has been shown to be associated with socioeconomic status and with income in particular [18]. Despite lower income inequality metrics in Japan, including egalitarian access to healthcare, the results of our study confirm the association between socioeconomic status and depression in Japan, as well.

Among health-related characteristics, although substance misuse has been suggested as externalizing factor of depression [46], neither current smoking status nor alcohol misuse were statistically significant in either bivariate or multivariate analyses. In contrast, having access to a PCP, prior diagnosis of depression and depression-associated diseases were associated with more self-reported depression in both bivariate and multivariate analyses. We suspect that these relationships are the result of reverse causality and suggest that primary care, a relatively less represented discipline of medicine in Japan, can play an important role in identifying individuals at risk for depression.

This study has several limitations that warrant discussion. First, the PHQ-9 was developed as a self-reported depression screening tool to assist clinical judgment and not as an actual diagnostic tool. Second, all depression screening variables and covariates were self-reported; a potential for bias exists, as previously reported in the literature [47]. However, we expect that any bias due to self-reporting will be largely non-differential misclassification, making our findings underestimates of the associations identified; we also assume the possibility of non-differential bias introduced by the financial incentive provided to participants in the sample panel. Third, though we found that depression-associated symptoms differed by age, the number and pattern of co-existing symptoms was not taken into account. As previous literature suggests that multiple somatic symptoms (i.e., 6 or more symptoms) are an independent predictor of depression and anxiety [48], future studies regarding the total number of simultaneously-occurring symptoms and their relationship to depression are warranted and may provide further clarity for early detection of depression. Fourth, we followed each individual for 1 month in a single year and, as a result, the results did not take yearlong frequencies and morbidities into account as several other factors related to season may influence health-seeking behaviors. In addition, for age-stratified analyses, though we used previously defined age categories (18–39, 40–65, and > 65 years old), these categories might not accurately distinguish all of the relevant life stages within each group, especially for the young adult cohort, into which both adolescents and adults were combined to maximize statistical power. Further study with different age categorizations in statistically appropriate numbers are warranted in order to further clarify the clinical characteristics attached to specific life stages. Finally, our analysis excluded those participants with incomplete PHQ-9 data (*n* = 34). The mean age for excluded individuals was 52 years old (38% men) versus 52 years old (47% men) in the analyzed sample. Although the gender distribution differed, given similar age distributions between incomplete group and total sample, and the relatively small (< 1%) number of incomplete data, we assumed an approximately equal frequency of errors.

## Conclusion

In conclusion, our study suggests that screening physicians may also require education on recognizing and treating age-related somatic correlates of depression: predominantly gastrointestinal symptoms in younger patients, and generalized fatigue, malaise, and musculoskeletal pain in older groups, implementing age-stratified strategies to detect the symptoms of depression. This study also reveals that the prevalence of PHQ ≥ 10 in Japan, while higher than previous estimates in Japan, is lower than western countries. Our data suggests that this discrepancy may be secondary to substantial underdiagnosis as only 13% of respondents with PHQ-defined at least moderate depression carried a diagnosis of depression. As untreated disease is associated with decreased quality of life and increased mortality, early detection of depression, including recognition of its somatic symptoms, is critical.

## Figures and Tables

**Fig. 1:**
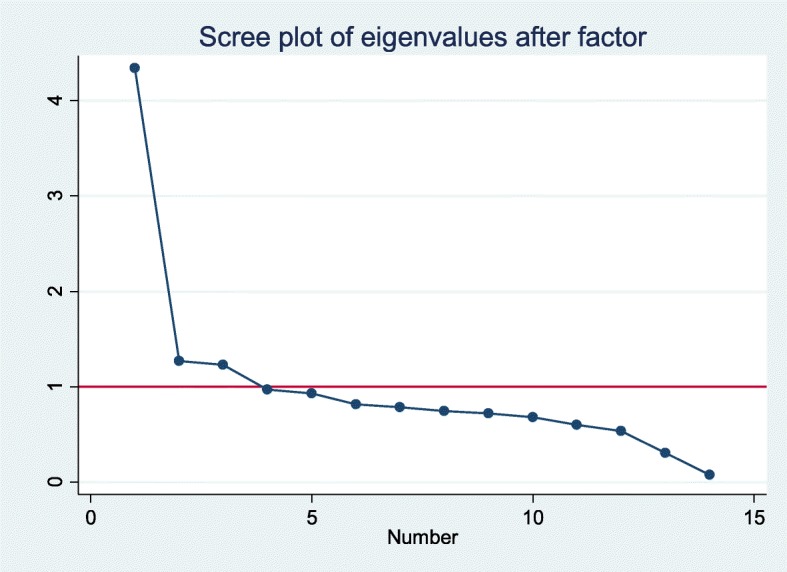
Scree plot

## Appendix

**Table 6:** Eigenvalues, coefficient alphas, and cumulative variance explained

Self-reported symptoms	Factor				
	1	2	3	4	5
neck and upper shoulder discomfort	.61				
shoulder pain	.57				
headache	.50				
low back pain	.47				
cough		.73			
fever		.63			
sore throat		.61			
generalised abdominal pain			.74		
diarrhea			.71		
fatigue and somnolence				.79	
general weakness and malaise				.70	
knee pain					.67
foot pain					.48
Eigen value	4.34	1.27	1.23	0.97	0.93
Cumulative variance explained	0.31	0.40	0.49	0.56	0.62

## Abbreviations

BMI: Body mass index
CI: Confidence interval
DSM-IV: Diagnostic and Statistical Manual of Mental Disorders- Fourth Edition
OR: Odds ratio
PCP: Primary care physician
PHQ-9: Patient Health Questionnaire
SD: Standard deviation
